# Near‐infrared spectroscopy in child and adolescent neurodevelopmental disorders

**DOI:** 10.1002/pcn5.59

**Published:** 2022-11-30

**Authors:** Kazuhiko Yamamuro

**Affiliations:** ^1^ Department of Psychiatry Nara Medical University School of Medicine Kashihara Japan

**Keywords:** attention‐deficit/hyperactivity disorder, autism spectrum disorder, near‐infrared spectroscopy, obsessive–compulsive disorder, Tourette's disorder

## Abstract

Near‐infrared spectroscopy (NIRS) is a noninvasive optical technique that uses the near‐infrared spectrum for functional neuroimaging by measuring oxygenation and hemodynamic changes in the cerebral cortex. The advantages of NIRS include its portability and ease of application, which allows for testing with the subject in natural positions, such as sitting or standing. Since 1994, NIRS has been increasingly used to conduct functional activation studies on different psychiatric disorders, most prominently schizophrenia, depression, bipolar disorder, and neurodevelopmental disorders. However, limited information on its use among child and adolescent patients is available. We herein review recent findings obtained using NIRS measurements of the brain during cognitive tasks in neurodevelopmental disorders, such as autism spectrum disorder, attention‐deficit/hyperactivity disorder, obsessive–compulsive disorder, and Tourette's disorder. This will facilitate evaluations of the causation and treatment of prefrontal cortex dysfunctions.

## INTRODUCTION

Neural activity near the brain's surface is noninvasively detected using multichannel near‐infrared spectroscopy (NIRS).^1^, ^2^ Near‐infrared light is used to assess changes in oxygenated hemoglobin (oxy‐Hb) and deoxygenated hemoglobin (deoxy‐Hb) concentrations in micro‐blood vessels on the surface of the brain. Local increases and decreases in oxy‐Hb and deoxy‐Hb concentrations have been shown to reflect cortical activity.^2^, ^3^ The combination of positron emission tomography (PET) and NIRS revealed a relationship between oxy‐Hb concentration changes and regional cerebral blood volume alterations.^4^, ^5^ NIRS is a suitable neuroimaging modality for evaluating psychiatric patients for the following reasons^6^: (i) examinations may be conducted in a natural sitting position without any surrounding distractions; (ii) the associated costs are markedly lower than those of other neuroimaging modalities; (iii) it is resistant to motion artifacts, and, thus, may be used in experiments in which motion occurs, such as vocalization by subjects; (iv) its set‐up is very straightforward; and (v) due to its high temporal resolution, NIRS can show the time course of prefrontal activity in psychiatric disorders.^7^, ^8^ Based on these advantages, NIRS is commonly applied to the examination of brain functions in many psychiatric disorders, including schizophrenia, depression, bipolar disorder, obsessive–compulsive disorder (OCD), posttraumatic stress disorder, dementia, autism spectrum disorder (ASD), and attention‐deficit/hyperactivity disorder (ADHD).

**Figure 1:**
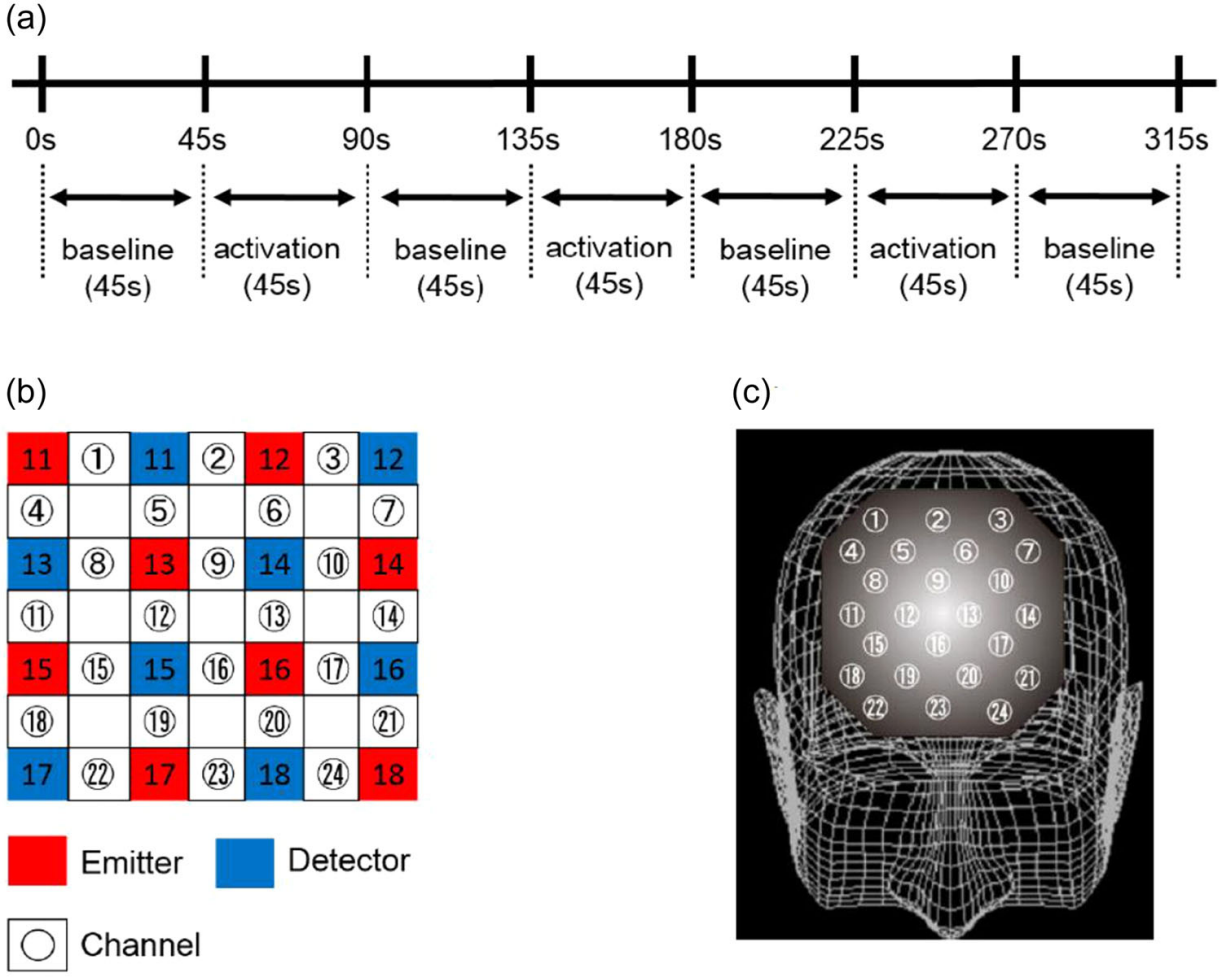
Location of 24 channels in the near‐infrared spectroscopy instrument. (a) Timeline of stimulus presentations of the Stroop color–word task. Patients read the words (blue, red, or green) printed on one page (baseline) and then named the ink color each word was printed in on the second page (activation). Each phase took 45 s, and this process was repeated three times. The baseline task was performed again for 45 s. (b) The arrangement of emitters and detectors corresponds to each channel. (c) The anatomical site of each track.

NIRS revealed that increases and decreases in oxy‐Hb and deoxy‐Hb concentrations reflect cortical activation. Since the direction of changes in deoxy‐Hb concentrations in animals was shown to be influenced by the extent of the changes in venous blood oxygenation and volume, regional cerebral blood flow may be accurately assessed based on changes in oxy‐Hb.^9^ Therefore, we evaluated oxy‐Hb using a 24‐channel NIRS machine (Hitachi ETG‐4000; Hitachi Medical Corporation), monitored the absorption of two wavelengths of near‐infrared light (760 and 840 nm), and the optical data obtained was assessed using a protocol that was performed according to the modified Beer–Lambert law,^10^ as previously described.^11^ Signals indicating changes in oxy‐Hb, deoxy‐Hb, and total‐Hb concentrations are calculated using this method. The quantity of hemoglobin measured is mmol × mm; therefore, all concentration changes depend on the path length of near‐infrared light. Recording channels are positioned in the optical path in the brain between neighboring pairs of emitters and detectors (Figure 1). The machine has an inter‐probe interval of 3.0 cm and can measure a point located 2–3 cm beneath the scalp (i.e., the surface of the cerebral cortex).^12^, ^13^

The focus of this review is to discuss current knowledge on the relationship between NIRS and the pathobiology of neurodevelopmental disorders, particularly in pediatric and adolescent patients. We examine ASD (ASD section; Table 1), followed by a focus on ADHD (ADHD section; Table 2), and then review previous studies on other disorders, such as OCD and Tourette's disorders (TD; Discussion section; Table 3).

**Table 1:** Autism spectrum disorder

Author	Participants	Age (years)	Task	Result
Child				
Uratani^19^	12 ASD patients, 12 controls	9.8 ± 2.3	Stroop color–word task	Smaller oxy‐Hb changes in the orbitofrontal PFC.
Kita^28^	10 ASD patients, 13 controls	10.2 ± 1.1	Self‐face recognition task	Oxy‐Hb changes were more significant in the right inferior frontal gyrus than in the left inferior frontal gyrus.
				Correlation between oxy‐Hb changes in the left inferior frontal gyrus and the severity of ADHD.
Iwanaga^31^	16 ASD patients, 16 controls	11.5 ± 1.8	MS task; Person's mental state	Smaller oxy‐Hb changes during the MS task in ASD patients.
			OC task; Object's characteristics	No significant differences in oxy‐Hb changes during the OC task between ASD patients and controls.
Funabiki^36^	11 ASD patients, 12 controls	16.8 ± 6.1	Attention to sound task	Cortical responses differed between groups in the prefrontal region but not in the auditory region.
Keehn^45^	27 infants at a high risk of autism (HRA), 37 low‐risk comparisons (LRA)	At 3, 6, 9, and 12 months		At 3 months, HRA infants showed increased overall functional connectivity.
				No significant differences were found between HRA and LRC infants at 6 and 9 months.
				At 12 months, HRA infants showed decreased connectivity.
Kikuchi^46^	15 ASD patients, 15 controls			ASD had significantly higher interhemispheric connectivity.
				No significant differences in low‐frequency fluctuations between groups.
				
				Correlation between higher connectivity and ADHD severity.
Adult				
Kuwabara^20^	10 PDD patients, 10 controls	26.5 ± 7.1	Letter fluency task	Smaller oxy‐Hb changes in the bilateral PFC.
Kawakubo^21^	27 ASD patients, 27 nonaffected siblings, 27 controls	Child group: 12.7 ± 3.4	Letter fluency task	No oxy‐Hb changes for children.
				Smaller oxy‐Hb changes in the bilateral PFC in adults
		Adult group: 26.7 ± 6.1		In adult siblings, the [oxy‐Hb] change was intermediate between those in controls and ASD patients.
Nakadoi^27^	14 PDD patients, 14 controls	31.6 ± 5.0	Fearful expression task	Smaller oxy‐Hb changes in the PFC.

Abbreviations: ADHD, attention‐deficit/hyperactivity disorder; ASD, autism spectrum disorder; MS, mental state; oxy‐Hb, oxygenated hemoglobin; PDD, pervasive developmental disorders; PFC, prefrontal cortex.

**Table 2:** Attention‐deficit hyperactivity disorder

Author	Participants	Age (years)	Task	Result
Child				
Negoro^60^	20 ADHD patients, 20 controls	9.55 ± 1.93	Stroop color–word task	Smaller oxy‐Hb changes in the bilateral inferior lateral PFC.
Weber^79^	11 ADHD patients, nine controls	10.4 ± 1.2	Trail‐making test	Controls showed an additional increase in deoxygenated hemoglobin in the left PFC.
				The ADHD group showed an increased tissue oxygenation index in the left PFC.
Yasumura^61^	10 ADHD patients, 15 controls	11.18 ± 2.23	Reverse Stroop task	Negative correlation between right LPFC activity and the severity of the attention deficit.
Inoue^63^	20 ADHD patients, 20 controls	9.58	Go/No‐go task	Smaller oxy‐Hb changes in frontal areas.
Xiao^64^	16 ADHD patients, 16 controls	9.75 ± 1.18	Go/No‐go task	Smaller oxy‐Hb changes in the right PFC.
Arai^67^	30 ADHD patients, 35 controls	9.5 ± 1.5	Spatial working memory task	Positive correlation between age and oxygenated hemoglobin in the frontal pole in controls, but not among ADHD group patients.
Ichikawa^73^	13 ADHD patients, 13 controls	9.75 ± 1.25	Happy and angry expressions	More significant variance in the peak latency of the hemodynamic response in the right temporal area.
Ishii^65^	18 ADHD patients, 27 controls	8.5 ± 1.4	Rock, paper, scissors task	Smaller oxy‐Hb changes in the DLPFC during the “to lose” task.
Adult				
Ueda^62^	12 ADHD patients, 12 controls	32.50 ± 9.13	Stroop color–word task	Smaller oxy‐Hb changes in the bilateral orbitofrontal cortex.
				Negative correlation between bilateral orbitofrontal cortex activity and ADHD symptoms.
Ehlis^66^	13 ADHD patients, 13 controls	29.8 ± 8.0	*n*‐back task	Smaller oxy‐Hb changes in the ventrolateral prefrontal cortex.
Drug				
Araki^81^	12 ADHD patients	9.8 ± 2.3	Continuous performance task	ATX induced improvements in right DLPFC activity after 6 or more months of the administration.
Ota^80^	10 ADHD patients	9.90 ± 2.38	Stroop color–word task	ATX induced improvements in bilateral DLPFC activity 8 weeks after the treatment
Matsuura^78^	11 ADHD patients	10.8 ± 1.8	Spatial working memory task	MPH induced improvements in PFC activity 4 weeks after the treatment.
Weber^79^	10 ADHD patients	10.7	Trail‐making task	MPH induced improvements in right PFC activity 4 weeks after the treatment.
Takahashi^87^	28 ADHD patients (drug naive; *n* = 20, drug nonnaive; *n* = 8), 20 controls	Drug naive: 8.6 ± 1.4	Stop signal task	Baseline NIRS signals, assessed after only MPH, showed that increased oxy‐Hb concentrations in the left inferior frontal gyrus predicted CGI‐S scores after a 4–8‐week or 1‐year MPH administration.
		Drug nonnaive: 9.3 ± 1.8		
Nakanishi^82^	30 ADHD patients (MPH; *n* = 16, ATX; *n* = 14)	MPH group: 8.19 ± 2.46	Stroop color–word task	Improvements in PFC activity 12 weeks later under the ATX condition.
		ATX group: 9.50 ± 2.03		No improvements in PFC activity 12 weeks later under the MPH condition.

Abbreviations: ADHD, attention‐deficit/hyperactivity disorder; ASD, autism spectrum disorder; ATX, atomoxetine; MPH, methylphenidate; MS, mental state; NIRS, near‐infrared spectroscopy; oxy‐Hb, oxygenated hemoglobin; PDD, pervasive developmental disorders; PFC, prefrontal cortex; TD, Tourette's disorder.

**Table 3:** Obsessive–compulsive disorder (OCD) and Tourette's disorder (TD)

Author	Participants	Age (years)	Task	Result
OCD				
Child				
Ota^99^	12 OCD patients, 12 controls	11.58 ± 2.07	Stroop color–word task	Smaller oxy‐Hb changes in the frontopolar cortex.
Adult				
Okada^98^	12 OCD patients, 12 controls		Stroop color–word task	Smaller oxy‐Hb changes in the left lateral PFC.
Hirosawa^100^	20 OCD patients, 20 controls	38.1 ± 10.9	Verbal fluency task	Smaller oxy‐Hb changes in the right DLPFC.
Liao^101^	70 OCD patients, 70 controls	27.6 ± 9.7	Verbal fluency task	Smaller oxy‐Hb changes in most areas of the prefrontal cortex.
Drug				
Abramowitz^102^	1 OCD patient	28	Verbal fluency task	After administering escitalopram (20 mg) for 6 months, it improved brain function in the temporal and frontal lobes.
TD				
Child				
Yamamuro^114^	10 OCD patients, ten controls	9.20 ± 2.25	Stroop color–word task	Smaller oxy‐Hb changes in the left DLPFC.

Abbreviations: oxy‐Hb, oxygenated hemoglobin; PFC, prefrontal cortex.

## ASD

Children with ASD have social impairments^14^ that may be partly attributed to an abnormality in self–other distinction.^15^ This abnormality was previously suggested to be responsible for some behavioral characteristics observed during self‐face recognition, a fundamental cognitive ability for social development.^16^ In 2014, the Centers for Disease Control and Prevention reported that nearly 1 in 68 children in the United States had ASD. Although the prevalence of ASD is rapidly increasing worldwide, its etiology has not yet been elucidated in detail.^17^ ASD is a highly heritable disorder, and interactions between susceptible genes and environmental factors have been proposed as a significant mechanism contributing to its etiology.^18^

Previous studies focused on functional neuronal activity in patients with ASD; however, limited information is currently available on this activity in pediatric and adult participants examined using NIRS during cognitive tasks. Brain activation during a Stroop color–word task (SCWC) was previously measured in 12 children with ASD and 12 healthy age‐ and sex‐matched children using NIRS, and oxy‐Hb changes in the dorsolateral prefrontal cortex (PFC) were found to be significantly smaller in ASD patients than in healthy controls.^19^

Kuwabara et al. examined 10 adult patients with pervasive developmental disorders (PDD) and 10 healthy age‐ and sex‐matched controls during a letter fluency task.^20^ Oxy‐Hb changes indicating specific activation in the bilateral PFC were significantly smaller in subjects with PDD than in control subjects. Consistent with these findings, another study revealed that NIRS signals showed substantially lower bilateral PFC activity during a letter fluency task in the adult ASD group (*n* = 27) than in controls; furthermore, in the adult siblings of ASD patients, the oxy‐Hb change was intermediate between those observed in controls and patients with ASD. However, there were no significant differences between oxy‐Hb changes in child ASD and control patients.^21^ These findings suggest the involvement of alterations in prefrontal activity in ASD development and genetic influences in these phenomena.

In healthy humans, face perception elicits activation within the occipitotemporal regions, including the fusiform face area (FFA).^22^ These areas are involved in processing facial expressions exhibiting emotion and the salient parts of the face. Perceptual information from these areas is sent to the amygdala and PFC, which are involved in appraising the emotional significance of stimuli and guiding social decisions and behavior. Studies on PDD using functional neuroimaging demonstrated hypoactivation of the FFA and amygdala in a face‐processing task.^23^, ^24^, ^25^ During face‐to‐face conversations, healthy controls' PFC and superior temporal sulcus (STS) were both found to be significantly activated. Furthermore, during face‐to‐face conversations, a negative correlation was observed between autism‐spectrum quotient (AQ) scores and regional cerebral blood‐volume increases in the left STS.^26^ These findings suggest that brain function, assessed using NIRS, is modified in ASD patients during realistic social interactions. Also, activation of the left STS during face‐to‐face conversations is weaker in typically developing participants with higher levels of autistic traits. A previous study in which 14 adults with PDD were compared with 14 typically developing children during a task on the implicit processing of fearful expressions showed significantly smaller changes in oxy‐Hb in the PFC in PDD patients than in healthy controls.^27^ However, children with ASD were found to have a specific capacity for self‐face recognition, a basic cognitive ability for social development.^28^ Brain activation was examined in 13 typically developing boys and 10 boys with ASD during self‐face recognition. Oxy‐Hb levels in regions corresponding to the right inferior frontal gyrus were higher than those in regions corresponding to the left inferior frontal gyrus. These levels were accurate indicators of the severity of ASD in groups of children with ASD; more severe ASD characteristics were associated with lower levels. Therefore, a dysfunction in the right inferior frontal gyrus area, which is responsible for self‐face recognition, may be an essential neural substrate contributing to the characteristics of ASD. This information may facilitate assessments of psychological aspects, such as public self‐consciousness.

The theory of mind and mental inference in ASD patients have been investigated in functional magnetic resonance imaging (fMRI) studies that assessed the capacity to understand feeling states and intentions, which generally emerges within the first 4 or 5 years of life and is crucial for social skills and progressing in the social world.^29^ Tasks involving images, stories, and animations designed to elicit the attribution of mental states were used. The findings obtained clearly demonstrated that the theory of mind in typically developing individuals are mediated by the posterior STS at the temporoparietal junction, temporal poles, amygdala, and dorsal medidal and ventrolateral prefrontal cortic.^30^ A previous study investigated brain activity in 16 children with ASD aged between 8 and 14 years and 16 age‐matched children.^31^ Oxy‐Hb concentrations in the prefrontal brain region were assessed using NIRS in tasks designed to show a subject's mental state (MS task) and an object's characteristics (OC task). The control group exhibited stronger activity than the ASD group, whereas significant differences were not observed between tasks (MS task vs. OC task) or hemispheres (right vs. left). NIRS revealed that when children with ASD performed MS tasks, activity was lower in the prefrontal brain area. Therefore, NIRS and these tasks may be used in clinical settings to detect brain dysfunctions in children with ASD related to the inference of mental states.

Behavioral and audiological studies demonstrated that behavioral responses to sounds in ASD patients were not associated with hypersensitivity of the auditory pathways but with issues in the higher cortical processing systems.^32^, ^33^ Electrophysiological findings indicated that sensory sound processing was intact, while involuntary orienting was affected,^34^ and that the early left frontal component, but not the temporal component, was abnormal.^35^ Collectively, these findings suggest that unawareness of sounds in ASD is not from a dysfunction in the auditory pathway itself. Consistent with these findings, changes in blood oxy‐Hb concentrations in the prefrontal and temporal cortices were measured using NIRS during various listening and ignoring tasks in 11 adolescent ASD and 12 control subjects.^36^ Oxy‐Hb concentrations in the auditory cortex increased with intentional listening, but not when the same auditory stimulus was ignored; this effect was observed in both groups. Cortical responses differed in the prefrontal region but not in the auditory region between the ASD and control groups. Thus, unawareness of sounds in ASD may be attributed to inattention rather than dysfunction in the auditory cortex.

Prospective longitudinal studies on infants at a high risk of developing ASD provide an insight into the earliest manifestations of these aberrant patterns of neurofunctional and structural connectivity. Atypical reductions in white matter connectivity have been reported in children with ASD and their siblings.^37^ The developmental trajectories of anatomical connectivity in high‐risk infants subsequently diagnosed with ASD were recently altered,^38^ indicating that atypical connectivity is an endophenotype or potential biomarker for ASD. Moreover, functional connectivity has been investigated using NIRS in typically developing infants^39^, ^40^ and adults.^41^, ^42^, ^43^, ^44^ Based on prospective longitudinal studies and NIRS, Keehn et al. examined functional connectivity in 27 infants at a high risk of autism (HRA) and 37 low‐risk comparisons (comparisons aged 3, 6, 9, and 12 months).^45^ At 3 months, overall functional connectivity was greater in water in HRA infants than in LRC infants. No significant differences were observed between HRA and LRC infants at 6 and 9 months. However, by 12 months, HRA infants showed less connectivity than LRC infants. These findings suggest that atypical functional connectivity exists within the first year of life, providing support to the growing body of evidence showing that abberrant pattarns of connectivity are a potential endophenotype for ASD. Furthermore, Kikuchi et al. demonstrated using a coherence analysis that inter‐hemispheric connectivity at 0.02 Hz was significantly higher in children with ASD (*n* = 15) than in controls (*n* = 15). In contrast, a power analysis did not reveal significant differences between the two groups at low frequencies (0.01–0.10 Hz).^46^ This aberrant higher connectivity in children with ASD positively correlated with the severity of social deficits, as scored using the Autism Diagnostic Observation Schedule. This study demonstrated aberrant brain functional connectivity between the right and left anterior PFC under conscious conditions in children with ASD.

## ADHD

With an estimated global prevalence of 3%–7%, ADHD is currently regarded as a neurodevelopmental disorder characterized by developmentally inappropriate attention, behavioral and cognitive impulsivity, and restlessness.^47^, ^48^ Inhibition difficulties in children with ADHD have been linked to prematurity, deficits in response selection, and motor adjustment.^49^ Hart et al.^50^ performed a meta‐analysis of functional imaging studies on children and adults; collectively, the findings obtained on inhibition revealed ADHD‐related hypoactivation in the right inferior frontal cortex, supplementary motor area, anterior cingulate cortex, and striatal‐thalamic areas, and ADHD‐related hypoactivation in the right dorsolateral PFC, posterior basal ganglia, and thalamic and parietal regions when focusing on attention tasks. Hart et al.^51^ Also, a meta‐analysis of timing tasks conducted by children and adults revealed ADHD‐related hypoactivation in the cerebellar vermis, left inferior PFC and insula, and left supramarginal gyrus that extended into the left superior temporal and postcentral gyri. Lee et al.^52^ performed single‐photon emission computed tomography (SPECT) scans were performed on children with clinical ADHD, demonstrating that orbitofrontal regional cerebral blood flow was decreased. fMRI also revealed the existence of dysfunctions in the PFC of children with ADHD.^53^, ^54^ SPECT scans on the orbitofrontal regions of children and adults with clinical ADHD also showed hypoperfusion.^55^, ^56^, ^57^ PET was also conducted on adult ADHD patients, and the findings demonstrated that glucose metabolism was reduced in the premotor and superior prefrontal cortices.^58^

Previous studies investigated functional neuronal activity levels in patients with ADHD, including pediatric and adult participants, during cognitive tasks using NIRS. Weber et al. examined 11 child patients with ADHD and nine healthy age‐ and sex‐matched controls and found lateralized oxygen consumption in the left PFC of normal controls during an extended‐attention task, whereas boys with ADHD showed an imbalance between oxy‐Hb and deoxy‐Hb during the short‐ and extended‐attention tasks.^59^ Negoro et al. subjected 20 children with ADHD and 20 healthy age‐ and sex‐matched children to NIRS to assess brain activation during SCWC.^60^ Changes in oxy‐Hb concentrations indicating specific activation in the inferior PFC were significantly smaller in children with ADHD than in the control group, and SCWC scores positively correlated with age. Consistent with these findings, another study comparing left and right hemisphere channels revealed significantly weaker right hemisphere activity in the child ADHD group (*n* = 10) during the reverse Stroop task.^61^ However, activity in the left hemisphere was not significantly stronger in the ADHD group than in the healthy (*n* = 10) and ASD (*n* = 11) groups. Furthermore, a negative correlation was observed between right lateral PFC (LPFC) activity and the severity of the attention deficit. Moreover, reduced oxy‐Hb in the bilateral orbitofrontal cortex was observed during SCWC in 12 adult patients with ADHD using 24‐channel NIRS.^62^ Furthermore, symptomatic severity negatively correlated with smaller changes in oxy‐Hb concentrations in the bilateral orbitofrontal cortex of the ADHD group.

Inoue et al. subjected 20 children with ADHD and 20 healthy age‐ and sex‐matched children to four‐channel NIRS to assess brain activation during a Go/No‐go task.^63^ They found that activation of the frontal areas during the condition with inhibitory demand (No‐go‐condition) was significantly less in children with ADHD than in typically developing children. However, the two groups noted no significant differences in activation during the condition without inhibitory demand (Go‐condition). Furthermore, activity levels in the right PFC were significantly lower in the child ADHD group (*n* = 16) than in the healthy group (*n* = 16) during the Go/No‐go task.^64^ One group performed tests on 18 children with AD/HD and compared the findings with 27 typically developing children.^65^ NIRS measured changes in oxy‐Hb levels in the prefrontal region during a “to lose” rock, paper, scissors (RPS) task. Children with ADHD showed significantly weaker oxy‐Hb activity in the prefrontal region during the “to lose” RPS task, particularly in the dorsolateral area. This finding indicated the potential of prefrontal region activation during the “to lose” RPS task, which is related to frontal inhibitory function, as a biomarker for diagnosing ADHD, which may facilitate the early treatment of ADHD.

Ehlis et al. examined 13 adult ADHD patients and 13 age‐ and gender‐matched healthy controls using multichannel NIRS while performing a working memory (*n*‐back) test.^66^ Task‐related increases in oxy‐Hb concentrations in NIRS channels located over the ventrolateral PFC were lower in ADHD patients than in the healthy control group, indicating reduced activation during the *n*‐back task performance in this part of the brain. These findings suggest working memory deficits and PFC dysfunctions in patients with ADHD. The hemodynamic activity was also examined in the PFC of 30 children with ADHD and 35 typically developing children while they performed a self‐generated spatial working memory (SWM) task.^67^ A negative correlation was observed with age and the number of errors in both groups (ADHD: *r*(28)  = −0.37, *p* = 0.040; typically developing children: *r*(33)  = −0.59, *p* < 0.001), indicating that self‐generated SWM improves through development. The typically developing group showed a positive correlation between age and oxy‐Hb in the frontal pole (10ch: *r*(33)  = 0.41, *p* = 0.013; 11ch: *r*(33)  = 0.44, *p* = 0.008) and bilateral lateral PFC (4ch: *r*(33) = 0.34, *p* = 0.049; 13ch: *r*(33) = 0.54, *p* = 0.001), while no correlation was observed in the ADHD group. Consistent with previous findings, children with ADHD showed abnormalities in the functional maturation of the frontal pole, which plays a role in manipulating and maintaining information associated with self‐generated behavior.

Children with ADHD have been reported to have other social cognitive impairments besides inattention, hyperactivity, and impulsivity. Although we still have limited knowledge on basic face processing in children with ADHD, the performance of these children and those with the most common comorbid disorder with ADHD, oppositional defiant disorder, in an emotional understanding task was significantly weaker than that of controls.^68^ The impaired emotional expression recognition was previously demonstrated in school‐aged children with ADHD.^69^, ^70^ Previous studies investigated recognition accuracy, using facial expressions of basic emotions, such as anger and happiness, in children with ADHD.^71^ The findings showed that ADHD children recognized angry faces less accurately but happy expressions as accurately as the controls.^70^, ^72^ Ichikawa et al. used NIRS to examine brain activation in the bilateral temporal area, which is sensitive to facial expressions, in response to happy and angry expressions, in 13 children with ADHD and 213 healthy age‐ and sex‐matched children.^73^ An increase was observed in oxy‐Hb concentrations for happy faces only in ADHD children but for both faces in the controls. Furthermore, variance in the individual peak latency of the hemodynamic response in the right temporal area was significantly greater in the ADHD group than in the controls. These findings suggest that atypical brain activation in boys with ADHD is associated with their preserved ability to recognize a happy expression but limited capacity to distinguish an angry face.

The dysregulation of prefrontal and subcortical catecholamine neurotransmission has been implicated in the pathophysiology of ADHD.^74^, ^75^ The most frequently prescribed drugs to treat ADHD are methylphenidate (MPH), a stimulant, and atomoxetine (ATX), a nonstimulant, both of which attenuate the clinical symptoms of ADHD. The mechanisms of action of both drugs involve modulating the neurotransmission of dopamine (DA) and norepinephrine (NE).^76^ Small changes in DA or NE concentrations affect networks of pyramidal cells in the PFC, which regulates and sustains attention.^77^ The therapeutic effects of these medications have been shown to occur in the PFC primarily^77^; however, the underlying mechanisms of action have not yet been elucidated in detail. Based on previous findings,^(^ ^73^, ^74^, ^75^, ^76^ ^)^ MPH and ATX are used in treating ADHD. A NIRS‐based investigation on medication assessed the effects of a clinical dose of MPH for 4 weeks on prefrontal hemodynamic responses in pediatric ADHD patients performing the SWM task.^78^ The findings showed that oxy‐Hb concentrations in the PFC were significantly higher in the post‐MPH condition than in the pre‐MPH state during a continuous performance task in 11 children with ADHD. In another NIRS study, Weber et al. showed that oxy‐Hb concentrations in the PFC were significantly higher in the post‐MPH condition than in the pre‐MPH state during a trail‐making task undertaken by 10 children with ADHD for 4 weeks.^79^ In contrast, changes in the prefrontal hemodynamic response during SCWC were investigated in pediatric ADHD patients treated with a clinical dose of ATX for 8 weeks^80^; ATX induced enhanced prefrontal hemodynamic responses in 10 child patients with ADHD. Araki et al. also demonstrated using NIRS that oxy‐Hb concentrations in the right dorsolateral PFC were significantly higher after treatment with ATX during a continuous‐performance task in 12 children with ADHD measured over 6 or more months.^81^ However, only one study directly compared MPH with ATX. Nakanishi et al. observed significantly greater changes in oxyhemoglobin concentrations in the PFC of participants treated with ATX than those at baseline (before the administration of ATX). However, we found no change, pre‐ and post‐treatment with MPH, in oxyhemoglobin concentrations.^82^ This variability in findings may be related to differences in cognitive tasks, dosages, patient ages, and treatment durations.

Approximately 30% of children with ADHD do not respond to MPH^83^; they show no benefit or only adverse side‐effects.^84^ A randomized study in which MPH and ATX were directly compared in adults with ADHD demonstrated that they exerted similar effects on executive functions; however, the findings indicated that ATX was slightly more effective at improving spatial planning than the immediate‐release formulation of MPH.^85^ In another head‐to‐head study that compared the two drugs, osmotically released MPH only improved set‐shifting and verbal fluency. In contrast, osmotically released MPH and ATX generally enhanced executive function in children and adolescents with ADHD.^86^ These findings suggest that ATX and MPH do not necessarily improve ADHD symptoms in patients with ADHD. Therefore, neuroimaging biomarkers to predict the clinical effects of the continuous administration of MPH are required and may be provided by NIRS. Takahashi et al. performed a double‐blind, placebo‐controlled, crossover trial with a single dose of MPH, followed by a prospective 4–8‐week open trial with continuous MPH administration and an ancillary 1‐year follow‐up.^87^ The findings found that baseline NIRS signals assessed during a stop‐signal task, measured just after taking only one 18‐mg MPH dose, revealed increased oxy‐Hb concentrations in the left inferior frontal gyrus that predicted the Clinical Global Impressions‐Severity score after the 4–8‐week or 1‐year administration of MPH.

## DISCUSSION

OCD is a mental disorder that affects 2%–3% of the general population.^88^ Recurrent, intrusive, and distressing thoughts and repetitive behaviors are the main clinical symptoms of OCD and significantly impair occupational and social functioning.^89^ Previous studies on patients with OCD using SPECT suggested the involvement of dysfunctions in the orbitofrontal cortex and caudate nucleus.^90^, ^91^ Furthermore, PET‐based studies on patients with OCD identified abnormally high functional activity in local brain regions, such as the orbitofrontal cortex, anterior cingulate cortex, and caudate nucleus.^92^ Several meta‐analyses also reported functional and structural abnormalities in the inhibition‐relevant areas of the brain, including the orbitofrontal cortex, anterior cingulate cortex, and caudate nucleus.^93^, ^94^ Moreover, the successful pharmacological or psychotherapeutic treatment of patients with OCD is associated with reductions in aberrant activity in the orbitofrontal cortex and caudate nucleus.^95^, ^96^, ^97^

Functional neuroimaging studies on OCD using NIRS reported dysfunctions in the PFC during executive tasks. Okada et al. examined neurobiological functions in 12 adult patients with OCD during SCWC. They found markedly smaller changes in oxy‐Hb concentrations in the left lateral prefrontal cortex in OCD patients than in 12 healthy control subjects.^98^ Their group investigated cerebral hemodynamic changes in response to SCWC in 12 OCD children and 12 healthy age‐ and sex‐matched controls. They found that oxy‐Hb changes in the control group were significantly larger than in the OCD group in the PFC, particularly in the frontopolar cortex, during SCWC.^99^ During a verbal fluency task (VFT), smaller changes in [oxy‐Hb] were noted in the right dorsolateral PFC of 20 adult patients with OCD by 42‐channel NIRS.^100^ Seventy adult patients with OCD and 70 age‐, gender‐, and education‐level‐matched healthy control subjects were also assessed while performing the VFT.^101^ Smaller changes were observed in oxy‐Hb concentrations in the PFC and temporal cortex, including the bilateral orbitofrontal cortex (OFC), right dorsolateral PFC, bilateral inferior prefrontal cortex (IPFC), bilateral frontopolar cortex (FPC), left superior temporal gyrus (STG), and bilateral middle temporal gyrus (MTG), in OCD patients than in the healthy control group. Furthermore, changes in oxy‐Hb concentrations in the right FPC negatively correlated with the Yale–Brown Obsessive–Compulsive Scale (Y‐BOCS) obsession score and Y‐BOCS total score. A negative correlation was also found between oxy‐Hb changes in the left OFC and the Y‐BOCS compulsions score.

Cognitive behavioral therapy (CBT) and selective serotonin reuptake inhibitors (SSRIs) are proven treatments for OCD.^102^ However, CBT is less commonly used than SSRIs.^103^ Moreover, approximately 60% of patients who have been treated with CBT did not receive the minimum criteria of CBT for adequacy. Exposure therapy in addition to escitalopram (20 mg) for 6 months was used to treat a 28‐year‐old female patient with OCD.^104^ NIRS revealed that this treatment improved brain function in this patient's temporal and frontal lobes. However, our group evaluated the brain activity of 12 pediatric patients with OCD during SCWC.^105^ Since patients in the OCD group were naive to treatment, we compared the findings obtained before and after 3 years of treatment with SSRIs. While SSRIs reduced OCD symptomology, they did not affect these patients' diminished hemodynamic responses or task performance. These findings suggest that a persistent deficit exists in the inhibitory control of pediatric patients with OCD.

The obsessive–compulsive symptoms of symmetry, ordering, aggression, religion, and sex are more commonly observed in TD patients than in those with primary OCD, while contamination is less frequent.^106^, ^107^, ^108^, ^109^ TD is a neuropsychiatric disorder that affects between 0.05% and 3% of children and is characterized by persistent motor and vocal tics. It often occurs with OCD, ADHD, and other social and behavioral disturbances; 50% of patients with TD also have ADHD, while 20%–60% have OCD.^110^, ^111^ Approximately 10% of patients only have TD.^112^, ^113^

Yamamuro et al. examined the cerebral hemodynamic changes during SCWC in 10 TD children and 10 healthy age‐ and sex‐matched controls.^114^ The findings showed that oxy‐Hb changes in the PFC during SCWC were significantly smaller in the TD group than in the control group, particularly in the left dorsolateral PFC. fMRI studies revealed functional abnormalities in TD patients' frontal and parietal areas. fMRI showed less neural activity in TD patients during spontaneous tic behavior,^115^ and resting‐state fMRI detected physiologically meaningful spontaneous low‐frequency (typically 0.01–0.1 Hz) fluctuations.^115^ Marsh et al.^116^ compared performance in the traditional Stroop task between children and adults with TD and healthy controls during fMRI data acquisition. More substantial behavioral Stroop interference effects were related to increased activation of the dorsolateral PFC and ventrolateral PFC in TD patients. Furthermore, tic severity scores positively correlated with dorsolateral PFC activation in TD patients, indicating an ineffectual compensatory mechanism. As described above, the present results were inconsistent with previous findings using a similar activation task. However, the present NIRS study included baseline and activation tasks, and relative oxy‐Hb concentrations were defined as the difference in oxy‐Hb concentrations between these tasks. Therefore, activation of the PFC was greater in the TD patient group than in control participants, even when the baseline task was conducted before the activation task. These findings may explain the significantly smaller differences observed in mean oxy‐Hb concentrations between the baseline and activation tasks in the TD patient group than in the control group. They also support the hypothesis linking a stronger prefrontal hemodynamic response during the Stroop task with TD in pediatric patients, as indicated by previous findings using imaging modalities, including fMRI and SPECT.

**Figure 2:**
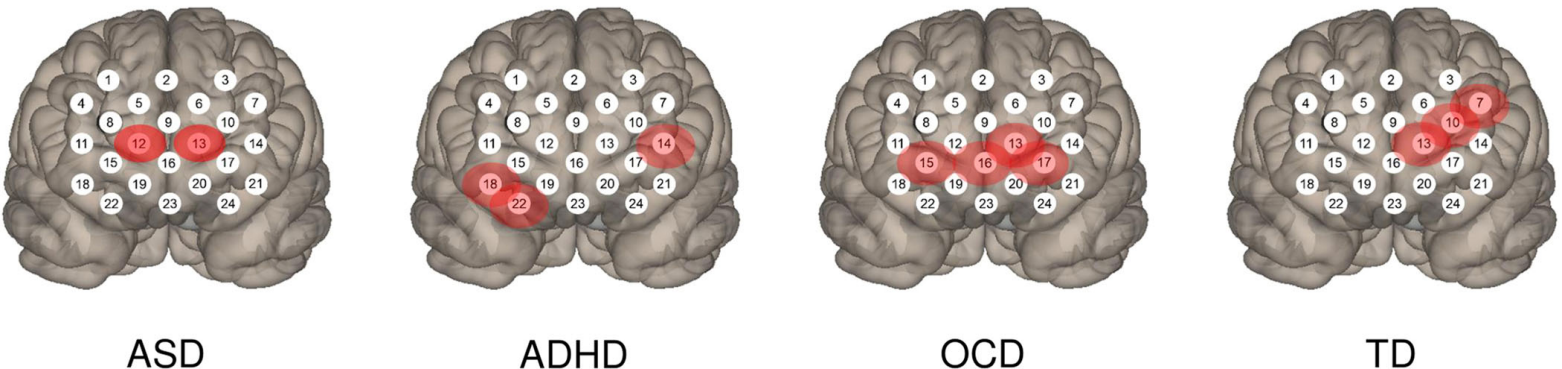
Differences between attention‐deficit/hyperactivity disorder (ADHD), obsessive–compulsive disorder (OCD), and Tourette's disorder (TD). Twenty‐four‐channel near‐infrared spectroscopy revealed differences in oxyhemoglobin concentrations during the Stroop color–word task among ADHD, OCD, and TD. Red indicates areas in which oxyhemoglobin levels were altered from those in healthy controls.

Consistent with these findings, previous studies showed smaller oxy‐Hb concentration changes in child and adolescent patients with neurodevelopment disorders than in healthy controls. However, these studies' activation tasks and age groups differed, which may have influenced changes in oxy‐Hb concentrations. Therefore, we attempted to examine differences in oxy‐Hb concentration changes among patients with various neurodevelopmental disorders considering activation tasks and age. Since SCWC performed by our group has been used in many neurodevelopmental disorder studies, we summarized the different brain regions in which SCWC reduced oxy‐Hb concentrations (Figure 2). The findings suggest that NIRS contributes to a differential diagnosis among various neurodevelopmental disorders; however, it is essential to note that NIRS imaging does not have high spatial resolution capabilities.

### Limitations

There are several potential limitations of NIRS measurements for patients with psychiatric disorders. First, NIRS has certain disadvantages compared with other methodologies, namely, that NIRS enables the measurement of Hb concentration changes as relative values only, not absolute values. We also asked the participants to perform the task three times and used an average. This was done to reduce the influence of potential accidental changes and reduce participant fatigue. Second, the spatial resolution for detecting hemodynamic responses from the scalp surface using NIRS is lower than that for fMRI, SPECT, and PET. However, abnormal prefrontal hemodynamic responses in individuals with several psychiatric disorders are certainly detectible by NIRS.

## CONCLUSION

Although NIRS is used in daily clinical practice in adult patients with depression to assist diagnoses in Japan, limited information is currently available to support the efficacy of NIRS imaging for diagnosing and monitoring children with neurodevelopmental disorders. However, NIRS may be highly advantageous in examining these disorders in early childhood because it is safe and convenient. Further evidence needs to be accumulated to support the conversion of this technique from a research tool to a standard clinical practice method for pediatric medicine.

## AUTHOR CONTRIBUTION

Kazuhiko Yamamuro conceived of and designed the review, and wrote the draft. Kazuhiko Yamamuro drew or made drafts of the figures. Kazuhiko Yamamuro has read and approved the final manuscript.

## CONFLICT OF INTEREST

The author declares no conflict of interest.

## ETHICS APPROVAL STATEMENT

The study protocol was approved by the appropriate ethics committees at Nara Medical University.

## PATIENT CONSENT STATEMENT

Not applicable.

## CLINICAL TRIAL REGISTRATION

Not applicable.

## Data Availability

Not applicable.
